# Nitrite mediated vasorelaxation in human chorionic plate vessels is enhanced by hypoxia and dependent on the NO-sGC-cGMP pathway

**DOI:** 10.1016/j.niox.2018.08.009

**Published:** 2018-11-01

**Authors:** Teresa Tropea, Mark Wareing, Susan L. Greenwood, Martin Feelisch, Colin P. Sibley, Elizabeth C. Cottrell

**Affiliations:** a Division of Developmental Biology & Medicine, School of Medical Sciences, Faculty of Biology, Medicine and Health, Maternal & Fetal Health Research Centre, University of Manchester, United Kingdom; b Clinical and Experimental Sciences, Faculty of Medicine, Southampton General Hospital and Institute for Life Sciences, University of Southampton, United Kingdom

**Keywords:** Pregnancy, Placental dysfunction, Nitrite, Nitric oxide, Hemoglobin

## Abstract

Adequate perfusion of the placental vasculature is essential to meet the metabolic demands of fetal growth and development. Lacking neural control, local tissue metabolites, circulating and physical factors contribute significantly to blood flow regulation. Nitric oxide (NO) is a key regulator of fetoplacental vascular tone. Nitrite, previously considered an inert end-product of NO oxidation, has been shown to provide an important source of NO. Reduction of nitrite to NO may be particularly relevant in tissue when the oxygen-dependent NO synthase (NOS) activity is compromised, e.g. in hypoxia. The contribution of this pathway in the placenta is currently unknown. We hypothesised that nitrite vasodilates human placental blood vessels, with enhanced efficacy under hypoxia.

Placentas were collected from uncomplicated pregnancies and the vasorelaxant effect of nitrite (10^−6^–5x10^−3^ M) was assessed using wire myography on isolated pre-constricted chorionic plate arteries (CPAs) and veins (CPVs) under normoxic (*p*O_2_ ∼5%) and hypoxic (*p*O_2_ ∼1%) conditions. The dependency on the NO–sGC–cGMP pathway and known nitrite reductase (NiR) activities was also investigated. Nitrite caused concentration-dependent vasorelaxation in both arteries and veins, and this effect was enhanced by hypoxia, significantly in CPVs (P < 0.01) and with a trend in CPAs (P = 0.054). Pre-incubation with NO scavengers (cPTIO and oxyhemoglobin) attenuated (P < 0.01 and P < 0.0001, respectively), and the sGC inhibitor ODQ completely abolished nitrite-mediated vasorelaxation, confirming the involvement of NO and sGC. Inhibition of potential NiR enzymes xanthine oxidoreductase, mitochondrial aldehyde dehydrogenase and mitochondrial *bc*_1_ complex did not attenuate vasorelaxation. This data suggests that nitrite may provide an important reservoir of NO bioactivity within the placenta to enhance blood flow when fetoplacental oxygenation is impaired, as occurring in pregnancy diseases such as pre-eclampsia and fetal growth restriction.

## Highlights

• Nitrite has been shown to induce vasorelaxation in a number of vascular beds, with enhanced efficacy under hypoxia.
• Here, we show that nitrite induces vasorelaxation also in isolated human placental blood vessels.
• Our data suggest nitrite vasorelaxation in placental blood vessels is via nitrite->nitric oxide reduction.
• The identity of the nitrite reductases responsible for the observed effects in this vascular bed remain unknown.

## 1 Introduction

Nitric oxide (NO) is a potent endogenous vasodilator important in the regulation of vasomotor tone, blood flow and systemic blood pressure [1]. In mammals, NO is synthesized by NO synthase (NOS) enzymes through sequential oxidation of one of the guanidino nitrogens of the amino acid l-arginine [2]. Although the inorganic anions nitrate and nitrite were previously believed to be physiologically inert oxidative end-products of endogenous NO metabolism, increasing evidence suggests that NO production from the reduction of nitrate/nitrite can be an alternative source of NO bioactivity in addition to the classical l-arginine—NOS—NO pathway [3].

It has been reported that orally ingested inorganic nitrate, abundant in green leafy vegetables and beetroot, is bioactivated following enterosalivary recirculation. Following absorption in the gut, a portion of the circulating nitrate is extracted by the salivary glands and actively secreted into saliva; in the oral cavity, bacterial nitrate reductases (NaRs) then reduce nitrate to nitrite. Upon swallowing, nitrite ends up in the acidic environment of the stomach where the corresponding nitrous acid (HNO_2_) decomposes to release NO and the remainder is re-absorbed as nitrite [3]. Circulating nitrite is taken up by blood vessels and other tissues and subsequently converted to NO-related products, which can be bioactivated to generate NO [4]. This is enhanced under conditions in which the physiological oxygen-dependent NOS enzyme activities are impaired [5], such as in hypoxia [6] or ischemia [7,8]. The importance of this ‘alternative’ pathway for NO production from nitrite has opened up a growing avenue for research where the use of nitrate/nitrite supplementation or nitrite delivery aims at improving NO bioavailability and bioactivity in diseases associated with local vasoconstriction and hypoxia.

Both enzymatic and non-enzymatic reduction of nitrite into NO have been proposed to occur within blood vessels and tissues [9]. Enzymes attributed with a NiR function include xanthine oxidoreductase [10,11], deoxymyoglobin [12,13], deoxyhemoglobin [14,15], mitochondrial aldehyde dehydrogenase [16], mitochondrial *bc*_*1*_ complex [6,17] and endothelial NOS itself [18,19]. The involvement of the classical NO-activation pathway via sGC and consequent elevation of cyclic guanosine monophosphate (cGMP) remains controversial, as does the significance of specific pathways of bioactivation [20], and likely depends on the tissue/vascular bed studied [4,8,10,11,21,22].

In contrast to systemic vascular beds, the fetoplacental circulation lacks innervation, and vascular resistance is completely dependent on structural and humoral factors, with NO being the key physiological vasodilator in this system [23]. At present, little is known regarding the effect of nitrite on the human fetoplacental vasculature [24]. It is plausible that nitrite may act as an important vasodilator and source of NO both in normal pregnancy, as well as in pregnancy complications associated with reduced uterine artery blood flow and oxygen delivery to the fetoplacental unit, such as pre-eclampsia and fetal growth restriction.

Herein we used wire myography to test the hypothesis that inorganic nitrite vasodilates human chorionic plate arteries (CPAs) and veins (CPVs) and that this effect is enhanced under conditions of hypoxia. Activation of the sGC pathway for nitrite-induced vasorelaxation and mechanisms for nitrite reduction were also investigated.

## 2 Materials and methods

This study was conducted with Research Ethics Committee approval (15/NW/0829), and in accordance with the Declaration of Helsinki. Informed consent was obtained from all women prior to tissue collection.

### 2.1 Samples

Placentas (n = 84) were obtained after elective Caesarean section following uncomplicated pregnancies (no evidence of hypertension, fetal growth restriction, diabetes or other medical disorders). Demographic details of study participants are reported in Table 1. Individualised birth weight centiles (IBCs) were calculated using the GROW Centile Calculator (v5.7.7.1, Gestation Network, www.gestation.net), which adjusts for: birth weight (g), parity at booking, maternal height (cm), booking weight (kg), ethnic origin, gestation (weeks/days) and baby gender. Biopsies were taken and placed directly into ice-cold physiologic salt solution (PSS; in mM, 117 NaCl, 25 NaHCO_3_, 4.69 KCl, 2.4 MgSO_4_, 1.6 CaCl_2_, 1.18 KH_2_PO_4_, 6.05 glucose, 0.034 EDTA; pH 7.4) within 30 min of delivery.

**Table 1:** Demographic details of study participants.

Demographic characteristics	Median (IQR)/number [%]
Maternal age, years	33 (30–36)
Pre-pregnancy maternal BMI, kg/m^2^	24 (22–27)
Maternal smoking	4 [4.8]
Maternal ethnicity	White/Caucasian 62 [73.8]
	Asian 12 [14.3]
	Black 4 [4.8]
	Other 6 [7.1]
Gestational age, days	273 (271–275)
Birth weight, g	3350 (3050–3617)
IBC, centile	51 (30–71)
Sex: Male	44 [52]

Data are shown as median and interquartile range (IQR; in parenthesis) or as number and percentage as appropriate. Abbreviations: BMI, body mass index; IBC, individualised birth centile.

### 2.2 Myography

CPAs and CPVs were identified macroscopically from the point of umbilical cord insertion as branches of the umbilical arteries and vein that continue to branch across the surface of the chorionic plate. Edge sections of the chorionic plate were selected. CPAs and CPVs of approximate resistance artery size (<500 μm), and therefore thought to contribute significantly to overall fetoplacental vascular resistance [25,26] were identified under a stereomicroscope, dissected free from the surrounding connective tissues and cut into ∼2 mm lengths. After mounting on two 40 μm steel wires in a myograph chamber (Model 620 M, Danish MyoTechnologies, Denmark), the vessels were immersed in 6 ml of PSS maintained at 37° C, gassed at two different oxygen tensions (see below) and normalized to an internal diameter of 0.9L_5.1kPa,_ in accordance with Mulvany's normalization procedure [27].

This procedure produces passive pressures of 2.4–3.4 kPa/18–26 mmHg [28], approximating the *in vivo* placental vascular pressure [29]. Following the normalization process, CPAs and CPVs were equilibrated for 20 min prior to the commencement of vasoactive studies. Vessels with diameters >500 μm were excluded from studies.

### 2.3 Induction of hypoxia

Experiments were performed in vessels normalized and equilibrated in 5%CO_2_/5% oxygen/90%nitrogen (to mimic the oxygen tension of the placenta under normal conditions, herein termed normoxia) or 5%CO_2_/95% nitrogen to reduce the oxygen tension of PSS (to mimic low placental oxygen, herein termed hypoxia). These conditions have been used previously to mimic normoxia and hypoxia [28] and result in 4.5–5.8% and 0.5–1.2% oxygen bath concentrations, respectively.

### 2.4 Functional experiments

Following equilibration, vessels were exposed to high potassium PSS (KPSS; 120 mM KCl in PSS, equimolar substitution of KCl for NaCl) to assess functional viability. Vessels that were unresponsive to KPSS (generating <0.1 mN/mm tension following KPSS application) were excluded from the study. Maximal vasoconstriction to KPSS was not affected by oxygen tension in either vessel types, but was significantly lower in CPVs compared to CPAs (data not shown). Vessels were then washed twice with 6 mL PSS to a stable baseline in order to restore the basal tone before being bathed a second time by KPSS. A dose-response curve to the thromboxane mimetic U46619 (10^−10^ - 2 × 10^−6^ M) was obtained and was used to calculate the EC_80_ concentration of U46619. Steady-state, sub-maximal preconstrictions with EC_80_ U46619 were obtained, and sodium nitrite (NaNO_2_) was then applied to the organ bath in a cumulative fashion (10^−6^ - 5 × 10^−3^ M at 2–5 min intervals). U46619 constriction responses replicated previous findings [28], with CPVs exhibiting significantly lower constriction compared with CPAs (see Supplementary Fig. 1). Constriction responses and U46619 EC_80_ doses were different between CPAs and CPVs, but neither was affected by oxygen tension (Supplementary Fig. 1, Supplementary Table 1).

In order to investigate mechanisms of vasorelaxation, in a separate series of experiments NaNO_2_ vasorelaxation was determined in normoxic CPAs, in the presence of the NO scavengers 2-4-carboxyphenyl-4,4,5,5-tetramethylimidazoline-1-oxyl-3-oxide (cPTIO, 1 mM) [30] and oxyhemoglobin (oxyHb, 20 μM; prepared according to Feelisch and Kubitzek) [31] and the sGC inhibitor 1H-[1,2,4]oxadiazolo [4,3-a]quinoxalin-1-one (ODQ, 10 μM) [11]. As oxyHb is oxidised over time, this was added to the organ bath every 20 min during the preconstriction/dose-reponse phases, along with an anti-foaming agent (SE-15; 100 μl) to minimize foam formation. In order to determine the role of potential NiR enzymes in mediating NaNO_2_-induced vasorelaxation, a further set of experiments was carried out using the following enzyme inhibitors (all applied 30 min prior to U46619 preconstriction): febuxostat (31.6 nM; nonpurine xanthine oxidoreductase-specific inhibitor) [11], N_ω_-Nitro-l-arginine methyl ester + N_ω_-Nitro-l-arginine (L-NAME, 100 μM + L-NNA, 100 μM; NOS inhibitors) [11], cyanamide (1 mM; mitochondrial aldehyde dehydrogenase inhibitor) [11,16] and myxothiazol (10 μM; mitochondrial *bc*_*1*_ complex inhibitor) [7]. All NiRs experiments were conducted under hypoxic conditions, when nitrite reduction to NO is proposed to be of key importance [9]. In parallel, both time and vehicle control conditions were assessed where appropriate but were not found to significantly affect vascular responses and thus for clarity are not shown.

### 2.5 Drugs and chemicals

Chemicals and pharmacological agents used in this study were purchased from Sigma Aldrich, unless otherwise stated. All compounds tested were applied to the organ bath from frozen small aliquots of stock solutions, except for NaNO_2_ and NOS inhibitors (Cayman Chemicals), stock solutions of which were prepared daily. NaNO_2_, cPTIO, cyanamide and NOS inhibitors were dissolved in PSS; myxothiazol in EtOH (final bath concentration 0.1%); ODQ (Cayman Chemicals) and febuxostat (Generon) in dimethyl sulfoxide (DMSO; final bath concentration 0.02% and 0.001%, respectively). All drugs were kept on ice in lightproof vials and further diluted in PSS as required.

### 2.6 Data analysis and statistics

Relaxation of vessels was expressed as a percentage change from the level of preconstriction achieved with an EC_80_ of U46619. All data are expressed as mean ± SEM and differences between groups analysed by two-way ANOVA followed by Tukey's post hoc test where appropriate. Differences in basal tone following oxyHb incubation were determined using Student's *t*-test. Statistical significance was defined as *P* < 0.05.

## 3 Results

**Fig. 1:**
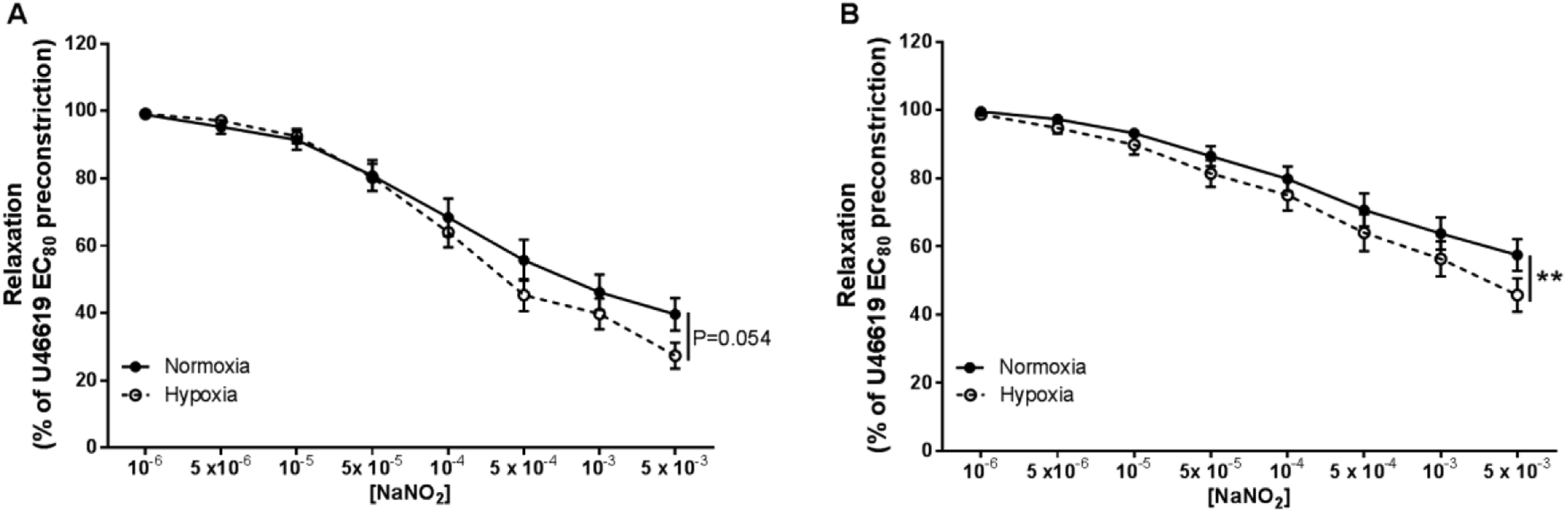
**Nitrite-mediated vasorelaxation of human chorionic plate vessels is enhanced in hypoxia.** Vasorelaxant effect of nitrite on CPAs (A) and CPVs (B) in normoxia and hypoxia. **P < *0.01,* normoxia vs hypoxia. n = 22–28 placentas per group.

In both CPAs and CPVs, cumulative addition of NaNO_2_ (10^−6^ – 5 × 10^−3^ M) caused a concentration-dependent vasorelaxation (Fig. 1 A, B). Under normoxic conditions, the maximal vasorelaxation of CPAs and CPVs was 38.9 ± 4.9% and 57.2 ± 4.6%, respectively (Fig. 1 A, B). Oxygen tension influenced the degree of vasorelaxation, as hypoxia enhanced nitrite's potency, significantly in CPVs (*P* < 0.01) and with borderline significance in CPAs (*P* = 0.054). In hypoxic conditions, the maximal vasorelaxation seen in CPAs and CPVs was 27.2 ± 3.8% and 45.6 ± 4.9% respectively, of maximal preconstriction to U46619 (Fig. 1 A, B).

**Fig. 2:**
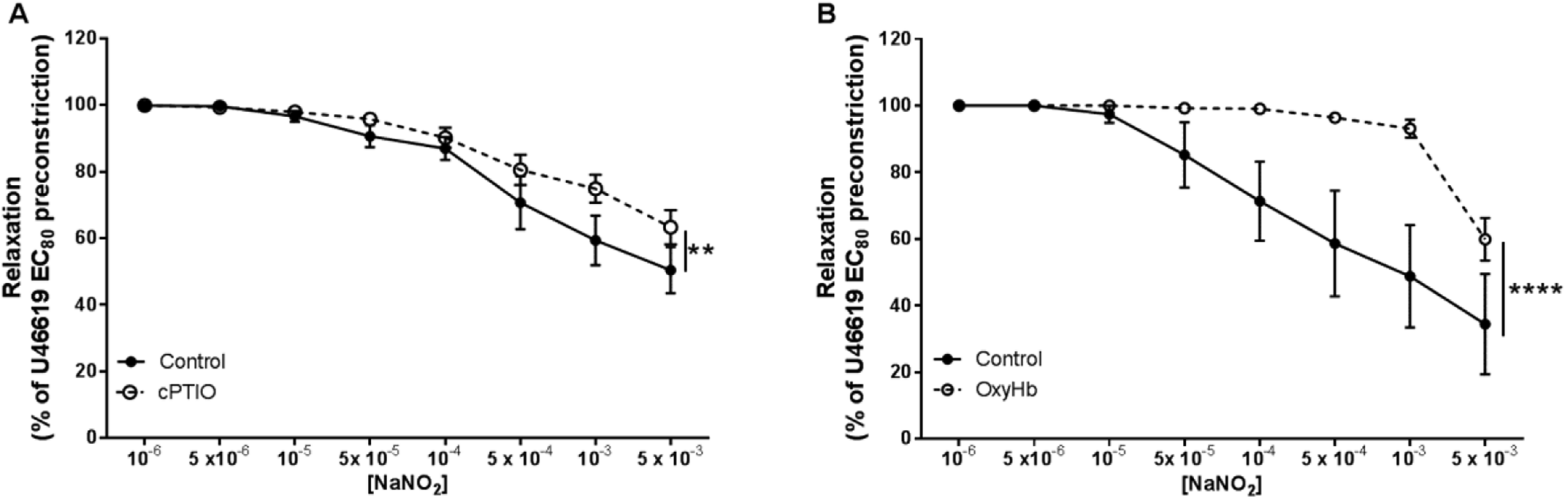
**Nitrite vasorelaxation of CPAs likely occurs via reduction to NO in conditions of normoxia.** (A) cPTIO and (B) oxyHb-mediated NO scavenging effect. **P < *0.01* control vs cPTIO; ****P < *0.0001* control vs oxyHb. n = 5–9 placentas.

The NO scavenger cPTIO, reduced the sensitivity of nitrite in CPAs, under normoxia (*P* < 0.01; Fig. 2 A). Following addition of oxyHb, a known NO scavenger, to the myograph baths, we noted an immediate vasoconstrictive effect, with vessels exhibiting a significant increase in basal tone to 0.81 ± 0.13 kPa, compared with 0.30 ± 0.07 kPa in control vessels (*P* < 0.01). Incubation with oxyHb largely abolished vasorelaxation to nitrite (*P* < 0.0001; Fig. 2 B). At the highest dose of nitrite (5 × 10^−3^ M), vasorelaxation was still observed.

**Fig. 3:**
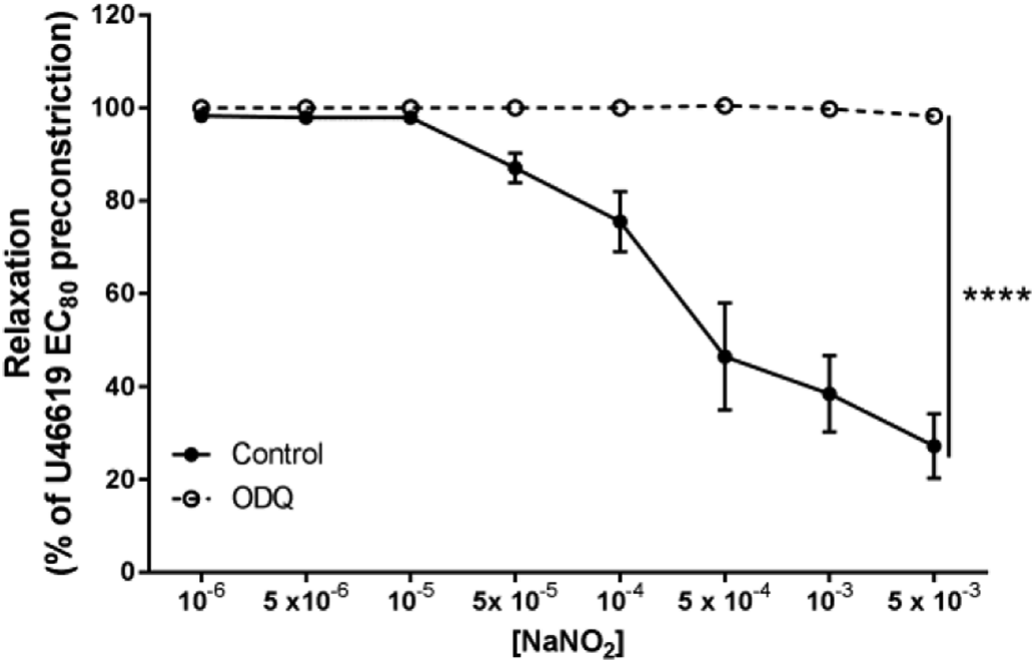
**Inhibition of nitrite vasorelaxation is abolished in the presence of ODQ in normoxic CPAs.** Role of sGC-cGMP signaling was investigated in CPAs preincubated with the sGC inhibitor ODQ. ****P < *0.0001* control vs ODQ. n = 6 placentas per group.

Additionally, we found that the vasorelaxant effect of nitrite was completely blocked by inhibition of sGC with ODQ (*P* < 0.0001; Fig. 3). Taken together, these data suggest that the classical NO-sGC-cGMP pathway is critically involved in nitrite-mediated vasorelaxation in human placental vessels.

**Fig. 4:**
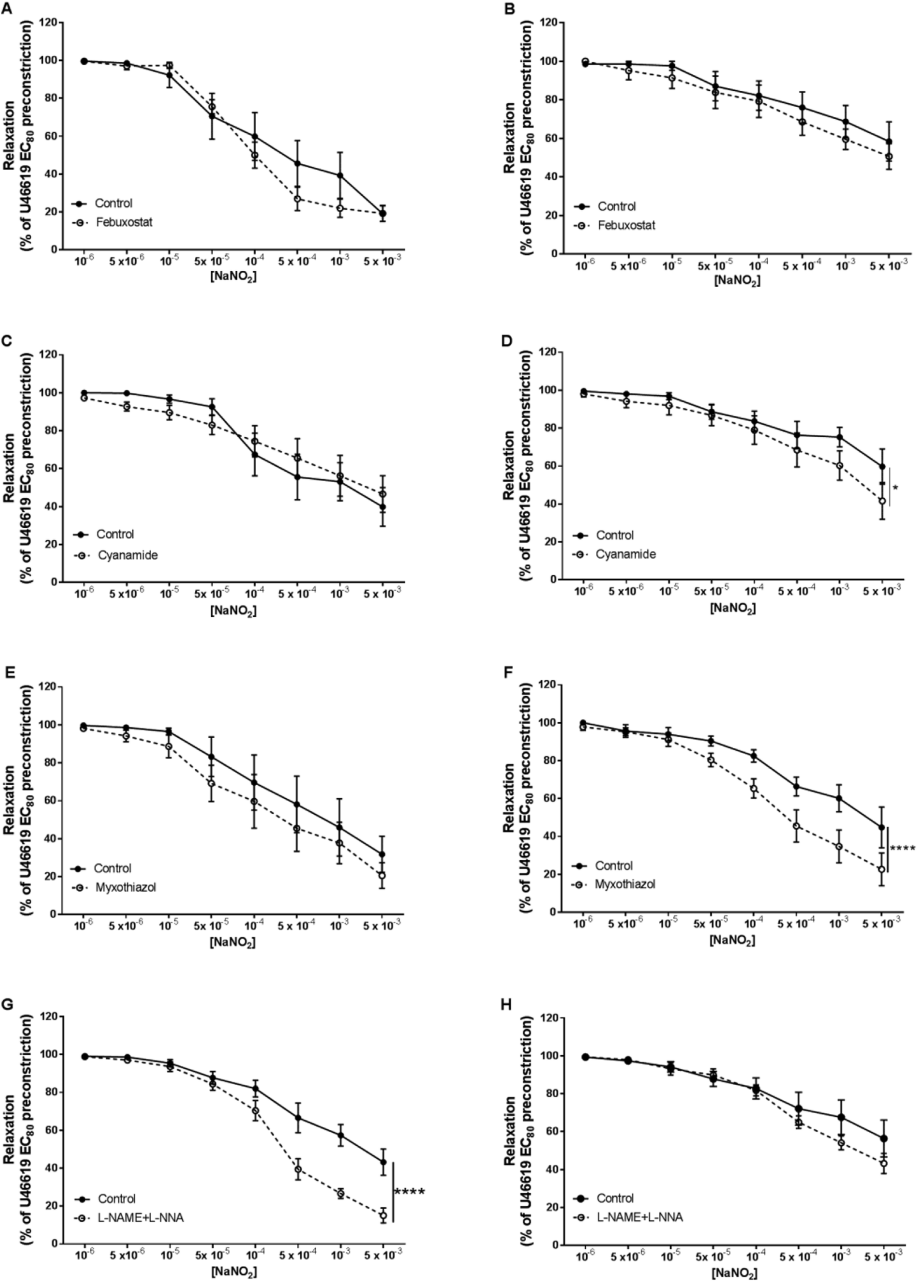
**Nitrite vasorelaxation of CPAs and CPVs is not dependent on known NiRs in conditions of hypoxia.** Inhibition of xanthine oxidoreductase with febuxostat in CPAs (A) and CPVs (B); inhibition of mitochondrial aldehyde dehydrogenase with cyanamide in CPAs (C) and CPVs (D); inhibition of mitochondrial *bc*_*1*_ complex with myxothiazol in CPAs (E) and CPVs (F); inhibition of NOS enzymes with L-NAME/L-NNA in CPAs (G) and CPVs (H). *P < 0.05, ****P < *0.0001*, inhibitor vs control vessels. n = 6 placentas for each group.

In order to investigate the potential mechanisms underlying NO generation from nitrite in isolated CPAs and CPVs, we used pharmacological inhibitors to target known NiR enzymes (Fig. 4). There were no effects of time or vehicle/inhibitor controls (DMSO, ethanol) on vascular responses (data not shown). Preincubation with the xanthine oxidoreductase inhibitor febuxostat did not alter vasorelaxation to nitrite in either CPAs or CPVs (Fig. 4 A, B). Interestingly, nitrite vasorelaxation was significantly increased in CPVs after inhibition of mitochondrial aldehyde dehydrogenase with cyanamide (*P* < 0.05; Fig. 4 D) and by inhibition of mitochondrial *bc*_*1*_ complex by myxothiazol (*P* < 0.0001; Fig. 4 F). In CPAs, preincubation with NOS inhibitors L-NAME/L-NNA also enhanced nitrite vasorelaxation (*P* < 0.0001; Fig. 4 G).

## 4 Discussion

The present study shows that 1) inorganic nitrite causes significant vasorelaxation in human chorionic plate vessels; 2) vasorelaxation is enhanced under conditions of low oxygen tension in CPVs; 3) vasorelaxation is attenuated by the NO-scavenging effect of oxyHb and abolished by sGC inhibition in CPAs and 4) nitrite reduction to NO does not occur via any of the candidate NiR enzymes investigated to date, including xanthine oxidoreductase, mitochondrial aldehyde dehydrogenase, mitochondrial *bc*_1_ complex or NOS enzymes. These findings suggest the involvement of the NO-sGC-cGMP signaling pathway in the vasorelaxatory actions of nitrite, however the mechanism(s) of nitrite reduction to NO in this vascular bed remain unclear.

Studies in healthy, non-pregnant human subjects infused with incremental doses of nitrite have demonstrated that the vasorelaxant effect elicited is marked in venous capacitance beds and moderate in resistance arteries [32]. In our experiments, nitrite was more effective in CPAs than CPVs. This could potentially reflect the differences in physiological oxygen tension between systemic and placental blood vessels. In the placenta, the partial pressure of oxygen reported is approximately 28 mmHg in veins and 16 mmHg in arteries [33]; the venous system carries oxygenated blood to the fetus, while the arteries carry relatively deoxygenated blood, which may explain the higher sensitivity to nitrite of the arterial bed. However, differences in smooth muscle cell populations and/or wall structure between arteries and veins can not be excluded.

In mammalian species, plasma nitrite levels are in the range of 0.1–1 μM [14,34,35]. In normal pregnant women plasma levels of nitrate/nitrite were reported to be in the 10 μM range in the umbilical blood vessels, with higher concentrations in umbilical arteries than in vein [36]. Our experiments required high concentrations of nitrite, similar to those reported in previous *in vitro* studies [24,37,38] to elicit vasorelaxation, as nitrite is a weak vasodilator due to very slow and inefficient chemical reduction to NO *in vitro.* However, levels of nitrite within tissues (e.g. aortic tissue) are reported to reach 10–100 μM [39]; concentrations at which we observed significant vasorelaxation in both CPAs and CPVs.

Attenuation of nitrite-induced vasorelaxation with the NO scavengers used here substantiates findings of previous studies in other vascular beds [10,13,15], which showed dependency of the vasorelaxation on NO generation. Although reportedly specific for NO, cPTIO attenuated but did not abolish vasorelaxation to nitrite under the experimental conditions used in the current study. It has been reported that the scavenging abilities of cPTIO may be impaired when treatments applied induce a gradual and continuous production of NO [40], which although not measured, is likely to be the case here. In contrast, oxyHb provided a more potent NO scavenging action. This large iron-containing protein produces a more effective NO sink that, in the present experiments, led to a near-complete abrogation of nitrite-mediated-vasorelaxation. The observed increase in basal tone supports the NO scavenging function of oxyHb, and suggests that constitutive production of NO from blood vessels in this *in vitro* system contributes to maintenance of vascular tone. Although oxyHb produced a more potent inhibition of nitrite-induced vasorelaxation, at the highest dose (5 × 10^−3^ M) vasorelaxation was still observed, probably due to saturation of oxyHb NO-scavenging capacity. This was associated with an observable colour change in the bath solution at this highest dose, from a red to brown colour (suggesting the formation of methemoglobin) and associated loss of scavenging function. Our data thus strongly suggest that nitrite effects on vasorelaxation are mediated via a conversion to NO, however to assert this conclusively, direct measurement of NO would be required.

The complete inhibition of vasorelaxation by the sGC blocker ODQ suggests a key role of NO-mediated activation of sGC, in line with several other studies [7,13,21,41]. It is also possible that nitrite might activate sGC directly, as has been shown previously [4]. However, the nature of interaction of NO and the role of sGC in mediating the vasorelaxant effect contrasts with studies reporting cGMP-independent signaling [10,11], that likely predominates in small size vessels such as in the renal microcirculation. Nitrite-mediated vasorelaxation is enhanced with increasing acidosis and hypoxia [42], both conditions in which the classical l-arginine/eNOS pathway is dysfunctional [5]; whereas the ‘alternative’ pathway is gradually activated due to dysinhibition by oxygen [6] to form bioactive NO through reduction of nitrite. As already mentioned, several potential enzymatic mechanisms have now been identified to reduce nitrite to NO. It has been reported that xanthine oxidoreductase is the functional NiR responsible for reduction of nitrite to NO in mouse renal arterioles [10] and interlobar arteries [11], in rat myocardium [43], in rat systemic [44] and pulmonary [45] vasculature. A role for mitochondrial aldehyde dehydrogenase and mitochondrial *bc*_1_ complex has been demonstrated in thoracic aorta, human gluteal subcutaneous fat resistance vessels [16] and in rat liver mitochondria [17], respectively. NOS enzymes have been found to act as NiRs in kidney [46]. We found that selective inhibitors of xanthine oxidoreductase, mitochondrial aldehyde dehydrogenase, mitochondrial *bc*_1_ complex and NOS enzymes did not block nitrite-induced vasorelaxation in CPAs or CPVs under conditions of hypoxia. On the contrary, we observed that inhibition of NOS in CPAs and mitochondrial aldehyde dehydrogenase and mitochondrial *bc*_*1*_ complex in CPVs increased nitrite-induced vasorelaxation; the former is in line with previous observations where sensitivity to nitrovasodilators increased following inhibition of basal NO production in the vasculature, which can be related to an up-regulation of sGC [47]. Mitochondria are one of the major sources of superoxide production, which impairs vascular smooth muscle relaxation by reducing NO bioavailability [48,49]. The enhanced vasorelaxation in CPVs pre-treated with inhibitors of mitochondrial aldehyde dehydrogenase and the mitochondrial *bc*_*1*_ complex may involve a mechanism that lowers superoxide generation, thus enhancing responses to nitrite-derived NO. In agreement with our data, studies in other vascular beds also reported that inhibition of xanthine oxidoreductase, mitochondrial *bc*_1_ complex [7], aldehyde oxidase, mitochondrial aldehyde dehydrogenase and NOS [11] were ineffective in inhibiting the nitrite response.

Although ODQ is widely believed to be a selective sGC inhibitor, it has been shown that this compound may also oxidise the heme groups of other proteins such as hemoglobin, myoglobin and cytoglobin [21,50], lacking of specificity for the sGC [51]. It is possible, therefore, that the complete abolition of nitrite-induced vasorelaxation in the presence of ODQ could involve not only sGC, but might also be due to an inhibition of the potential NiR function of vascular globins, as there is a growing body of evidence that the heme globin family can function as NiRs in a number of tissues [13,14,21,52].

## 5 Conclusions

Our study demonstrates that nitrite vasodilates human placental blood vessels via production of NO, and that this response is potentiated under conditions of low oxygen availability. Nitrite reduction to NO may play an important role in the normal physiological regulation of vascular tone in the fetoplacental circulation, although the mechanisms underlying nitrite reduction remain to be determined in this vascular bed. Therapeutic interventions that increase the ‘reservoir’ of nitrite may potentially be of benefit in compromised pregnancies and of relevance for placental vascular dysfunction, to ensure sufficient NO production with maintenance of fetoplacental vascular tone and adequate nutritional supply of the fetus.

## Conflicts of interest

All authors declare no conflicts of interest.
