# Supplementary material for: Nitrite mediated vasorelaxation in human chorionic plate vessels is enhanced by hypoxia and dependent on the NO-sGC-cGMP pathway
Source: Nitric Oxide. 2018 Nov 1;80:82–8. doi: 10.1016/j.niox.2018.08.009 (PMC6199414; doi:10.1016/j.niox.2018.08.009)
Supplement: Supporting Information [file mmc1.docx]

**Supplementary data**

**

**

**Supplementary Fig. 1. U46619-induced vasoconstriction of CPAs and CPVs from human placentas.** CPAs generate significantly greater tension in response to U46619 compared with CPVs. Hypoxia did not alter U46619 responsiveness in either vessel types. ****P<0.0001, CPAs vs CPVs. Data are presented as active effective pressure, expressed in kPa. All data are mean ± SEM. n= 84 placentas.

**Supplementary Table 1**

**U46619 EC80 doses in human placental vessels.**

| U46619 | Log EC_80_ (mol/L) | |
| --- | --- | --- |
| Vessel type ^a^ | Normoxia | Hypoxia |
| CPAs | -6.944 ± 0.018 | -6.938 ± 0.015 |
| CPVs | -6.862 ± 0.041 | -6.904 ± 0.034 |

Log EC_80_, logarithm of the molar concentration of U46619 causing 80% of the maximal vasoconstriction prior to nitrite dose-response curves were calculated. CPVs required a significantly higher concentration of U46619 to achieve EC_80_ preconstriction compared with CPAs (^a^ P <0.05, CPAs vs CPVs). There was no significant effect of oxygen tension on U46619 EC_80_ concentration in either vessel types. All data are mean ± SEM. n=84 placentas.

**Supplementary Table 2**

**Vasorelaxation to nitrite in human placental vessels.**

| **Experimental condition** | **Log EC_50_ (mol/L)** | **Vmax (%)** |
| --- | --- | --- |
| **Nitrite** | **Normoxia**  **Hypoxia** | **Normoxia**  **Hypoxia ^a^** |
| CPAs  CPVs | -3.798 ± 0.13 -3.769 ± 0.115  -3.715 ± 0.138 -3.729 ± 0.146 | 38.88 ± 4.924 27.18 ± 3.77  57.16 ± 4.563^c^ 45.61 ± 4.855^c^ |
| **Normoxia** | **Control cPTIO** | **Control cPTIO** |
| CPAs | -3.438 ± 0.251 -3.199 ± 0.166 | 49.97 ± 6.885 63.28 ± 5.135 |
| **Normoxia** | **Control OxyHb** | **Control OxyHb** |
| CPAs | -3.465 ± 0.267 n. m. | 34.43 ± 15.11 59.86 ± 6.415 |
| **Normoxia** | **Control ODQ** | **Control ODQ** |
| CPAs | -3.598 ± 0.201 n. m. | 27.25 ± 6.984 98.22 ± 1.028^d^ |
| **Hypoxia** | **Control Febuxostat** | **Control Febuxostat** |
| CPAs | -3.736 ± 0.407 -4.065 ± 0.095 | 19.1 ± 4.174 18.01 ± 4.2 |
| CPVs | -3.374 ± 0.461 -3.806 ± 0.441 | 56.77 ± 10.23 50.64 ± 6.66 |
| **Hypoxia** | **Control Cyanamide** | **Control Cyanamide** |
| CPAs | -3.718 ± 0.238 -3.638 ± 0.236 | 39.13 ± 10.17 46.62 ± 9.686 |
| CPVs | -3.794 ± 0.372 -3.252 ± 0.21 | 59.67 ± 9.298 41.57 ± 9.653 |
| **Hypoxia** | **Control Myxothiazol** | **Control Myxothiazol** |
| CPAs | -3.296 ± 0.496 -3.722 ± 0.3 | 31.75 ± 9.504 20.48 ± 6.771 |
| CPVs | -3.472 ± 0.148 -3.695 ± 0.141 | 44.75 ± 10.71 22.64 ± 8.667 |
| **Hypoxia** | **Control L-NAME + L-NNA** | **Control L-NAME + L-NNA** |
| CPAs | -3.476 ± 0.2 -3.622 ± 0.106 | 43.17 ± 6.914 15.06 ± 3.978^b^ |
| CPVs | -3.729 ± 0.354 -3.537 ± 0.145 | 56.37 ± 9.727 43.25 ± 5.339 |

Log EC_50_ (logarithm of the molar concentration of each drug causing 50% of the maximal relaxation), and Vmax, (maximum relaxation), for nitrite-mediated vasorelaxation in each experimental condition (see Results in the main text). Relaxation of vessels was expressed as a percentage change from the level of preconstriction achieved with an EC_80_ dose of U46619. ^a^P < 0.05 hypoxia vs normoxia; ^c^P < 0.001 CPAs vs CPVs; ^d^P < 0.0001 ODQ vs control; ^b^P < 0.01

L-NAME+L-NNA vs control; n.m., not measurable. All data are mean ± SEM.
